# The Optimal Fibular Strut Bone Graft Fixation Angle for Unstable Proximal Humerus Fractures: A Finite Element Analysis

**DOI:** 10.3390/bioengineering12101078

**Published:** 2025-10-03

**Authors:** Hyun Seok Song, Hui-Gyeong Gong, Hyun-Ju Lee, Hyungsuk Kim, Ki-Sik Tae

**Affiliations:** 1 Department of Orthopedic Surgery, Eunpyeong St. Mary’s Hospital, College of Medicine, The Catholic University of Korea, Seoul 03312, Republic of Korea; hssongmd@catholic.ac.kr; 2 Department of Biomedical Engineering, Konyang University, 158 Gwanjeodong-ro, Daejeon 35365, Republic of Korea; 23606007@konyang.ac.kr; 3 Department of Physical Therapy, Konyang University, 158 Gwanjeodong-ro, Daejeon 35365, Republic of Korea; leehj@konyang.ac.kr

**Keywords:** proximal humerus fracture, locking plate fixation, fibular strut graft, finite element analysis

## Abstract

Adding a fibular strut bone graft to locking plate fixation has been introduced to improve stability and prevent varus collapse. The purpose of this study was to perform finite element analysis (FEA) of the biomechanical characteristics of different insertion angles of the fibular strut graft in proximal humerus fractures. Proximal humerus fractures with metaphyseal comminution and instability were simulated by creating wedge-shaped osteotomies medially and laterally for varus and valgus models, respectively. Three-dimensional finite element models were reconstructed from computed tomography images. A locking compression plate with a length of 90 mm (three holes) was applied to the proximal humerus fracture model. Fibular allografts were inserted at 0° and 30° to the humeral shaft. Axial and traction forces of 70°, 90°, and 110° relative to the vertical axis were applied to each model to simulate stress on the plate and graft. At axial loads, stresses in both the plate and the graft were lower when the graft was inserted at 0° than at 30°. Under traction loads, plate stress was lower with 30° insertion. Graft stress was also lower with 30° in most experimental conditions in both the valgus and varus models. These findings suggest that oblique insertion may provide biomechanical advantages under traction forces in unstable proximal humerus fractures.

## 1. Introduction

Surgical treatment of displaced proximal humerus fractures is technically demanding, especially when the fracture involves the medial column with comminution [1,2]. Failure to reconstruct medial support is a significant risk factor for fixation failure and can lead to varus collapse followed by screw penetration, eventually resulting in implant failure [3,4,5].

The incidence of proximal humerus fracture was reported as the 3rd most common fracture after distal radius and hip fractures in populations older than age 65 [6,7]. In the United States, the adult incidence is about 60 per 100,000, while among older populations the rate is substantially higher—for example, approximately 250 per 100,000 in Medicare beneficiaries, and in individuals aged ≥ 70 years 424 per 100,000 in women and 150 per 100,000 in men [8]. In Europe, population-based studies report incidences of 60.1 per 100,000 person-years and 82 per 100,000 person-years, with higher rates in women and a sharp rise with age [9]. Most proximal humerus fractures in the elderly arise from low-energy falls and occur in osteoporotic or osteopenic bones. Poor bone quality compromises inferomedial support, leading to complex fracture patterns with comminution and displacement and, after fixation, increasing the risk of loss of reduction, varus collapse, and screw cut-out.

To overcome these difficulties with poor bone quality, locking plate fixation with medial support screw, also known as calcar screw or inferomedial screw, has shown satisfactory clinical results [10]. However, the most common complications of locking plate fixation include screw cut-out and varus collapse [3]. Initial varus fracture alignment is reported to increase complication rates [11]. Restoration of the medial column is the key factor in achieving successful treatment for complex proximal humerus fractures [12].

Adding a fibular strut bone graft to locking plate fixation was introduced not only to improve stability but also to prevent varus collapse [13]. Biomechanical studies with both synthetic and cadaveric bone have reported that the strut graft increased failure load and overall stiffness of the bone-implant construct, which reduced migration of the fracture fragment [14,15]. In addition, multiple clinical studies reported satisfactory results using locking plates with strut bone graft [4,16,17,18].

To the best of our knowledge, there is a lack of studies comparing the insertion angle of the fibular bone strut graft. Gardner et al. [13] first introduced fibular strut bone graft by inserting the strut bone vertically into the humeral canal and inserting a push screw to push the fibula medially to reduce the medial column. Most of the studies described the same principles [18,19,20]. However, the longer fibular graft could not reach the subchondral bone of the inferomedial head even when pushed medially.

Neviaser et al. [19] described the varus and valgus fracture of proximal humerus fixation with fibular strut graft. In varus unstable fracture, the fibula was placed along the calcar and tamped into the medial subchondral bone of the humeral head. In contrast, the fibula was kept laterally to fill the void left by the disimpacted humeral head component in valgus unstable fractures [16,21].

Finite element analysis (FEA) has been increasingly applied in orthopedic biomechanics to evaluate implant performance and stress distribution under physiological loading. Unlike cadaveric or synthetic bone experiments, FEA provides standardized conditions, reproducibility, and the ability to assess various loading scenarios in detail [22,23,24]. These advantages make FEA a useful method for investigating fixation stability in proximal humerus fractures and the mechanical role of fibular strut grafts.

The purpose of this study was to compare the biomechanical characteristics of fibular strut grafts in different fixation angles in varus and valgus unstable proximal humerus fracture using a finite element analysis (FEA). We hypothesized that fibular strut graft inserted obliquely would provide more stability in varus unstable fracture and graft inserted vertically in valgus unstable fracture.

## 2. Materials and Methods

The research protocol was approved by the Institutional Review Board (IRB) of Eunpyeong St. Mary’s Hospital (Approval No. PC22ZISI0018). Given the retrospective nature of this study and the use of anonymized imaging data from a single patient, the requirement for informed consent was waived by the IRB.

### 2.1. Finite Element Models (FEM)

DICOM images were obtained from a computed tomography (CT) scan (SOMATOM Definition Edge, Siemens Healthineers, Forchheim, Germany) of a 73-year-old male. The images were converted into and reconstructed as a three-dimensional (3D) finite element model (FEM) using MIMICS (Materialise Interactive Medical Image Control System, version 21, Materialise NV, Leuven, Belgium) software.

**Figure 1:**
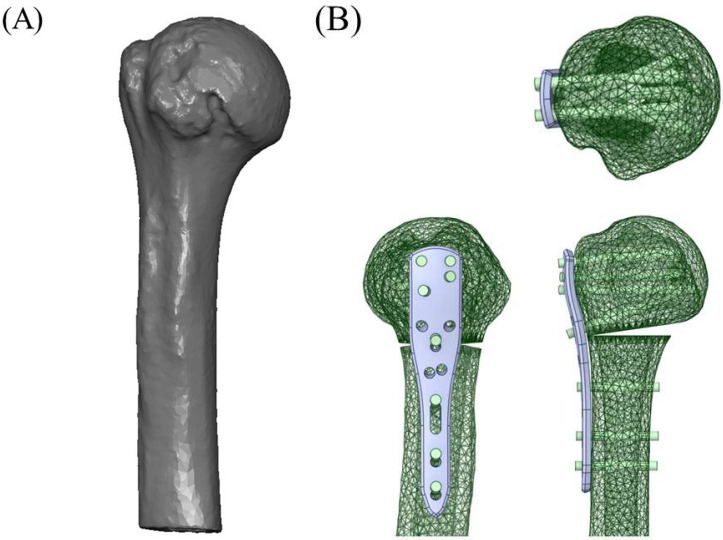
(**A**) Three-dimensional proximal humerus model reconstructed from computed tomography data of a 73-year-old male. (**B**) Varus unstable fracture model created by a medial wedge-out osteotomy with a locking compression plate and screws applied according to standard surgical guidelines.

A locking compression plate (PHILOS^®^ system; DePuy Synthes, Oberdorf, Switzerland) with a length of 90 mm (3 holes) was applied to the proximal humerus fracture model. Locking screws with lengths of 50, 48, 45, and 30 mm were applied to the head and three cortical screws with lengths of 30 mm were applied to the shaft. The threads of the screws were omitted to simplify the models. All models were simulated using SolidWorks 2015 (Dassault Systèmes, Waltham, MA, USA). The plate was positioned on the model according to standard surgical guidelines [10] (Figure 1).

**Figure 2:**
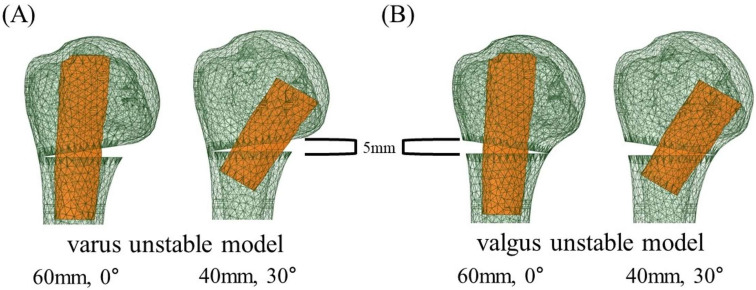
Fibular strut graft insertion with 0° and 30° angles to the humeral shaft. (**A**) Varus unstable model with graft inserted at 0°. (**B**) Valgus unstable model with graft inserted at 30°.

Proximal humerus fracture with a metaphyseal comminution and instability was simulated by creating a wedge-shaped osteotomy at the medial side (medial wedge-out) for varus unstable model and at the lateral side (lateral wedge-out) for valgus unstable model. The length of the removed bone was 5 mm. To make the proximal and distal fragments independent for the experiment, a 0.2-mm microdefect was created at the thin end of the wedge. Fibular allografts of 60 mm and 40 mm in length were inserted in at angles of 0° and 30° to the shaft of the humerus, respectively (Figure 2).

### 2.2. Finite Element Assessment (FEA)

The finite element assessment was carried out using ANSYS Workbench 21.2 (Ansys Inc., Canonsburg, PA, USA). The total number of nodes in the finite model ranged from 68,200 to 91,500, depending on the fibular graft insertion angle.

All bone and graft materials were modeled as linear elastic isotropic materials. The elastic modulus of the proximal humerus was set at 13,800 MPa with a bone mineral density (BMD) of 0.83 g/cm^3^, while the fibular graft was assigned an elastic modulus of 17,000 MPa and a BMD of 2.13 g/cm^3^. A Poisson’s ratio of 0.30 was applied to both the humerus and the fibular graft models [25]. The plate and screws were modeled as Ti-6Al-4V titanium alloy with an elastic modulus of 110 GPa and Poisson’s ratio of 0.34, also assumed to be linear elastic and isotropic.

Meshing was performed using 10-node quadratic tetrahedral elements (ANSYS SOLID187), which were applied uniformly to all solid components (bone, graft, plate, and screws) to ensure compatible interpolation across contact interfaces. A mesh convergence study was performed (coarse–medium–fine meshes), and the medium mesh (~30,000–45,000 elements) was selected after confirming that further refinement produced a <5% change in peak stress values [15].

The friction coefficient between proximal humerus and fibular graft was 0.2 [26]. Contacts between plate and screw and screw and bone were defined as fully fixed [27]. Contact elements in the study were defined as deformable elements. For boundary conditions, the distal cut surface of the humerus was fully constrained, with all translational and rotational degrees of freedom fixed, to simulate experimental clamping of the distal humerus. Loads were applied to the humeral head through a reference point using remote force coupling. A constant force magnitude of 500 N was used in all simulations to isolate the effect of traction direction and graft insertion angle on stress distribution. This magnitude lies within ranges reported in previous biomechanical and FEA studies of the proximal humerus [15,28].

Axial and traction forces were applied to each model. A total of four conditions were set, comprising two types of osteotomized model (varus and valgus unstable models) with two types of fibular graft insertion angle (0° and 30°). Traction forces were applied at 70°, 90°, and 110° relative to the vertical axis, representing approximately 20° abduction, neutral, and 20° adduction positions of the shoulder joint, respectively. These angles fall within the physiological range of glenohumeral joint reaction force directions reported in in vivo studies [15,28,29].

**Figure 3:**
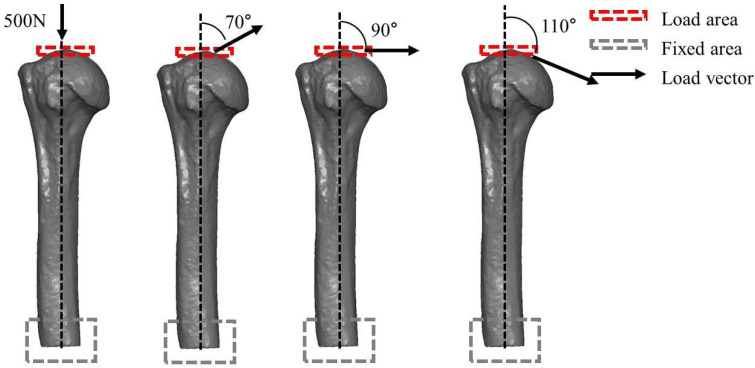
Loading conditions applied in the finite element analysis. Axial compression and three traction forces at 70°, 90°, and 110° relative to the vertical axis were applied to the humeral head to simulate rotator cuff traction in different abduction angles. Each force was set at 500 N.

For plate, screws and humerus, von Mises stress was calculated. For the fibular graft, which are brittle materials, maximum principal stress was computed to better represent fracture risk (Figure 3).

High von Mises stress observed on the plate was interpreted as a great risk of plastic yielding and fatigue cracking that may result in metallic failure. Dispersion of stress was interpreted as a positive effect in preventing implant failure. The maximal principal stress observed on the fibular graft was assessed to determine how the strut graft functioned as a buttress and how it contributed to the distribution of applied forces under each condition.

## 3. Results

### 3.1. Von Mises Stress in the Plate and Maximum Principal Stress in the Fibular Graft in the Varus Unstable (Medial Wedge-Out) Model

When axial load was applied, von Mises stress on the plate and the maximum principal stress in the fibular graft were higher at 30° than at 0° insertion. Under traction loads (70°, 90°, and 110°), plate von Mises stress was lower at 30° across all angles, and graft maximal principal stress was also lower at 30° at 90° and 110°; the only exception was 70° traction, where graft maximal principal stress was higher at 30° (Table 1).

### 3.2. Von Mises Stress in the Plate and Maximum Principal Stress in the Fibular Graft in the Valgus Unstable (Lateral Wedge-Out) Model

Higher stresses were observed in the 30° insertion model under axial loading—plate von Mises stress and graft maximal principal stress were both higher than at 0°. When traction force was applied at different angles, plate von Mises stress was lower at 30° at all angles, and, in contrast to the axial condition, the graft’s maximum principal stress was also lower at 30° at all traction angles (Table 2).

### 3.3. The Von Mises Stress Distribution on Contact Surface

**Figure 4:**
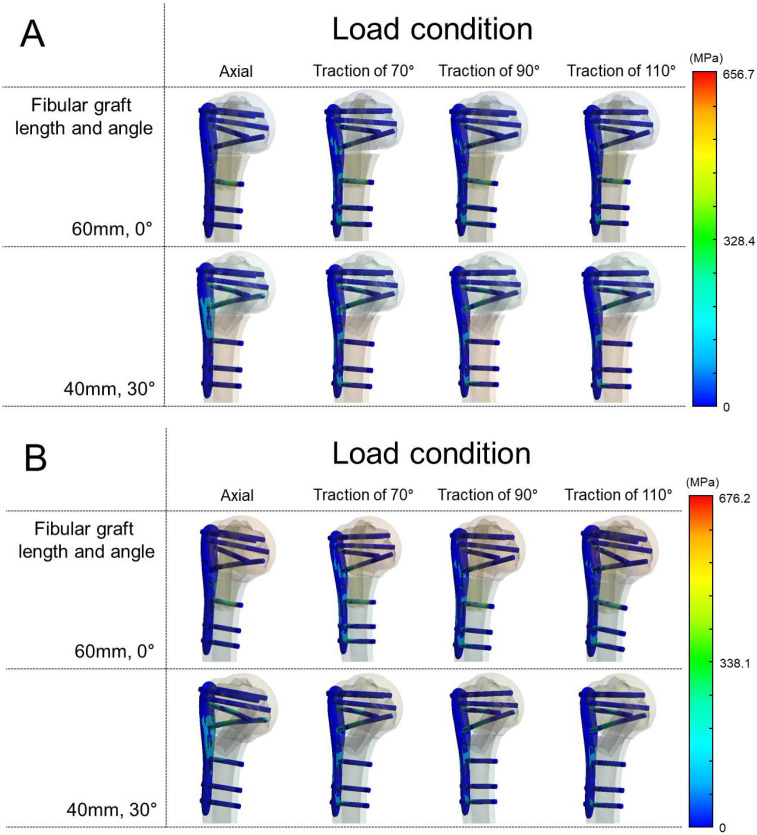
Von Mises stress distribution in screws. When the fibular graft was inserted at 0°, stress concentration occurred at the most proximal screw in both varus and valgus unstable models. (**A**) Varus unstable model. (**B**) Valgus unstable model. A color scale bar indicates von Mises stress (MPa).

**Figure 5:**
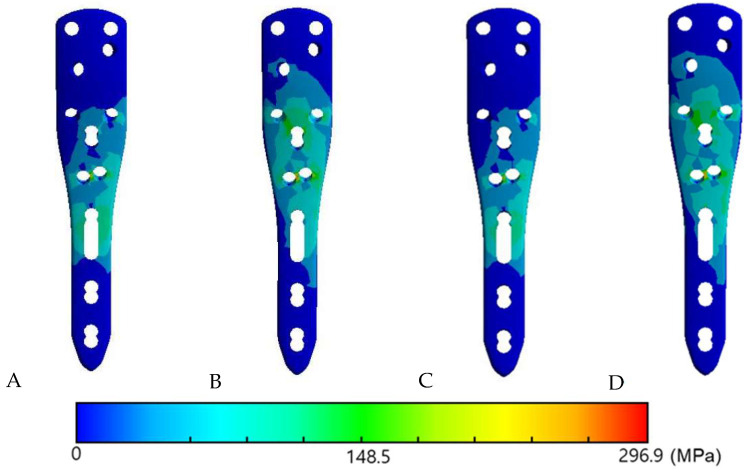
Von Mises stress distribution of the plate under traction loads. (**A**) Varus unstable model with fibular graft inserted at 0°. (**B**) Varus unstable model with graft inserted at 30° showing more dispersed stress distribution on the screw–bone interface. (**C**) Valgus unstable model with graft inserted at 0°. (**D**) Valgus unstable model with graft inserted at 30° showing more dispersed stress distribution compared with 0°. A color scale bar indicates von Mises stress (MPa).

When the fibular graft was placed at 0°, stress concentration was observed on the most proximal cortex screw in both varus and valgus unstable models under axial load (Figure 4). On the other hand, under all angles of traction force other than axial load, a dispersed stress distribution on the screw–bone contact surface was observed when the graft was placed at 30° in both varus and valgus unstable models compared to when the graft was place at 0° (Figure 5).

Additionally, the maximum von Mises stresses of the screws themselves were analyzed. Under axial loading, screw stresses were relatively low (138–166 MPa). In contrast, traction loads (70°, 90°, and 110°) markedly increased the stresses, reaching 495–676 MPa. In all simulations, the highest stress was consistently observed in screws traversing the fibular graft. In the 0° models, both the proximal 4th and distal 1st screws penetrated the graft, and the distal 1st screw exhibited the greatest stress concentration. In the 30° models, however, the distal 1st screw did not engage the graft, and the proximal 4th screw instead showed the highest stress. The numerical results of the maximum screw stresses are summarized in Table 1 and Table 2.

Finally, the maximum von Mises stresses at the humeral plate–bone interface are also summarized in Table 1 and Table 2. Under traction loads (70°, 90°, and 110°), these values were generally higher at 30° than at 0°.

## 4. Discussion

The major finding of this study is that, excluding axial loading, oblique (30°) insertion yielded lower plate von Mises stresses across traction directions (70°, 90°, and 110°) in both varus and valgus unstable models compared with vertical (0°) insertion. For the fibular graft (brittle), the maximum principal stress was also lower with oblique insertion in five of six traction scenarios, with the varus–70° condition as the only exception. Under axial loading, both plate stress and graft σ_1_ were higher at 30° than at 0°. Moreover, when traction force was applied in this study, stress was widely distributed on the plate and screw in both varus and valgus unstable models when the fibular graft was inserted obliquely.

Fixation failure with varus collapse is particularly disappointing in proximal humerus fractures, especially under pathologically osteoporotic conditions. Medial column support is essential when treating varus unstable fractures with medial column comminution. Locking screws in the humeral head provide angular stability, resulting in higher resistance to failure. However, the complication rate with locking plate is reported to reach 36–49% including malreduction, screw penetration and loss of fixation [30,31]. Such complications can necessitate revision surgery or conversion to arthroplasty [32,33]. These considerations align with our findings under traction, in which oblique insertion reduced plate stresses in both instability patterns.

Gardner et al. [34] suggested the use of an oblique locking screw in the inferomedial region of the head with locking plates. An oblique locking screw in the inferomedial region, commonly referred to as calcar screw or inferomedial screw [21,35], provides a counteracting force to the varus deforming force and consequently reduces the risk of varus collapse. Use of this screw has shown a positive effect on clinical outcomes, including complication rates, functional scores, and reduction loss [36,37]. A biomechanical study by Zhang et al. [38] reported that the oblique locking screw (as medial support) enhanced mechanical stability and prevented implant failure. Consistent with this rationale, our FEA indicates that oblique placement of the fibular graft can function as an inferomedial buttress analogous to calcar support: across traction directions, plate stresses were consistently lower with oblique insertion, and the graft’s maximum principal stress was lower in five of six traction scenarios; the only exception occurred in the varus–70° condition. These patterns support a load-sharing mechanism through the inferomedial column rather than the plate alone.

Valgus angulated proximal humerus fractures, on the other hand, are characterized by maintenance of the posteromedial periosteum, commonly referred to as medial hinge [12]. Preservation of the medial hinge not only helps with reduction of the fracture but also helps to avoid avascular necrosis (AVN) of the humeral head [39,40]. Due to these anatomic characteristics, treatment options range from conservative treatment and percutaneous fixation to locking plate fixation. After reducing the valgus-impacted humeral head, use of bone grafts, including a fibular bone strut graft, is an acceptable option for filling the lateral void [41]. In our models, the buttress effect of an obliquely inserted fibular graft was evident in both varus and valgus instability: under traction loads at 70°, 90°, and 110°, oblique insertion reduced plate stresses in both patterns and lowered the graft’s maximum principal stress across all traction angles in valgus, with similar reductions in most traction scenarios in varus. At the humeral plate–bone interface, peak von Mises values tended to be higher with oblique insertion under traction, which is consistent with load sharing through the bone–implant junction and is interpreted as a relative indicator of interface loading rather than a fracture threshold.

Using an intramedullary strut graft was first introduced by Walch et al. [42] for treating nonunion of proximal humerus fractures. It was first named the intramedullary bone peg when using the iliac crest [43], anterior tibial crest or fibula. Then, Gardner et al. [13] reported the technique of inserting the strut fibular bone vertically into the humeral canal and then pushing the fibula medially to support the medial column by inserting a push screw. Mechanical support of the medial column was provided by inserting a fibular graft endosteally and the fibular graft itself aided in reduction. A biomechanical cadaveric study by Bae et al. [14] demonstrated that locking plate fixation with strut graft augmentation significantly increased both the maximum failure load and initial stiffness compared to locking plate fixation alone. Several other studies reported prevention of varus collapse and AVN with improved patient-reported outcomes through the use of a fibular strut graft [16,18,19]. Myers et al. [44] reported that using fibular strut graft also preserved the head shaft angle on plain radiographs without increasing surgical time or morbidity. Extending this literature, our FEA directly compared insertion angles and showed that, under traction loading, oblique (30°) insertion reduced implant stresses and lowered the graft’s maximum principal stress in most scenarios.

There is a lack of studies on the optimal insertion angle of the fibular bone strut graft. Gardner et al. [13] first introduced the fibular strut bone graft by inserting the strut bone vertically into the humeral canal and then inserting a push screw to push the fibula medially to reduce the medial column. Most studies described the same principles [18,19,20]. However, the longer fibular graft could not reach the subchondral bone of the inferomedial head even when pushed medially. Tan et al. [20] also adopted this approach, placing the fibular graft vertically into the intramedullary canal.

Neviaser et al. [19] and Little et al. [17] placed the fibular graft differently depending on the location of the comminution. In the case of a varus unstable fracture, the fibula was positioned in the intramedullary canal first and placed along the medial arch. In a valgus unstable fracture, the fibular graft was positioned laterally to function as a buttress for the humeral head or to fill the void left by the disimpacted humeral head component.

We agree with the concept of Neviaser et al. [19] and Little et al. [17], and the purpose of this study was to evaluate it using FEA. Except for axial loading, oblique (30°) insertion lowered plate von Mises stresses across traction loads at 70°, 90°, and 110° in both varus and valgus unstable models. For the fibular graft, assessed by maximum principal stress, oblique insertion reduced stresses across all traction angles in the valgus model and in most traction angles in the varus model. Under traction, stress concentration on the plate was more widely distributed with oblique insertion in both models.

Clinical outcomes are quite promising with a small number of complications according to previously reported studies with both vertical and oblique insertion of the fibular graft. Matassi et al. [18] reported 100% radiographic healing without major complications, including AVN, humeral head collapse or screw cut-out, in their 17 patients by placing the fibular graft medially. Myers et al. [44] reported a lower incidence of complications, including coronal collapse (5%), AVN (6.5%), screw breakage or loosening (6.6%) and revision surgery (1.7%), in the group using fibular strut graft vertically. Tuerxun et al. [45] reported a lower complication rate in the group with fibular allograft placed vertically (7.3%) compared to the locking-plate-alone group (27.3%). Neviaser et al. [19] reported only one case of partial AVN and one case of loss of reduction was reported in 38 patients by placing the fibular graft vertically or obliquely depending on whether the fracture was varus or valgus unstable.

Technically, the longer fibular graft could not reach the subchondral bone of the inferomedial head even when pushed medially. In our study, grafts longer than 40 mm could not be placed obliquely. The fibular graft was inserted at 30°, similar to the in vitro biomechanical study by Osterhoff et al. [15].

Defect models in prior biomechanical work have varied, including horizontal segmental defects of 5–10 mm [28,46,47,48], ladder-shaped osteotomy with an unstable medial column [49], and medial gaps [29,38,50]. In this study, wedge-out osteotomies were created laterally or medially, and FEA was performed in both varus and valgus unstable models. Loads used in the literature include axial compression, shear, rotational, and traction. Many proximal humerus models have relied on axial compression [14,19,46], whereas others angled the construct to mimic abducted shoulder loading [28,29,51], and Osterhoff et al. [15] simulated rotator-cuff tension at 45–60° abduction. Although axial compression is common in experimental setups, it does not reflect physiological shoulder loading dominated by multidirectional muscle–joint force vectors [15,28,29,51]; therefore, the interpretation emphasized traction directions at 70°, 90°, and 110°. Under these traction conditions, oblique 30° insertion lowered plate stresses in both varus and valgus models and reduced the graft’s maximum principal stress in valgus across all traction angles, with similar reductions in most angles in varus.

Loads applied to the biomechanical model can include axial compression, shear, rotational, and traction. Most studies of proximal humerus fracture models have used axial compression loads [14,19,46]. In the study by Yang et al. [28] load was applied to the articular surface with the model inclined at 52.5° to the vertical. The authors described that this load could replicate physiological loads at the proximal humerus in 90° abduction. In the studies of Fletcher et al. [29,51] three different loads (45° abduction with 0° internal rotation, 45° abduction with 45° internal rotation, and 45° flexion with 0° internal rotation) were applied. In the biomechanical study of Osterhoff et al. [15] the tensile forces of the rotator cuff were simulated at 45–60° abduction. However, the proportion of each load to the shoulder during daily activities could not be clarified. In our study, axial and traction force at 70°, 90° and 110° to vertical axial force were applied to each model. These angles corresponded to 20° abduction, 0° abduction, and 20° adduction of the shoulder, respectively.

Cortical bone is a brittle material; using maximum principal stress provides a more accurate representation of fracture risk than von Mises stress. The high local principal stress values observed in the graft are still below reported ultimate tensile strength values for cortical fibula [35,36,37]. Nevertheless, these local stress concentrations highlight the importance of proper screw placement and graft seating to avoid iatrogenic fracture.

The strength of this study is the direct comparison of 0° and 30° insertion angles in varus and valgus unstable models across multiple traction directions. We suggest that oblique insertion of the fibular strut graft may be an optimal option to sustain multidirectional distraction forces after fracture reduction and fixation, particularly under traction loading.

However, there were some limitations in this study. First, this study used CT data from one elderly patient, so the results may not apply to the general population. We selected CT data from an elderly patient to better reflect osteoporotic bone conditions. Second, because the FEA was based solely on the bone, effects of muscles, tendons, and ligaments were not considered; to partially address this, traction loads were applied at three directions. Third, a constant load magnitude of 500 N was applied across all simulations to isolate the effects of load direction and insertion angle. Although this value is supported by prior biomechanical and finite element studies, actual joint and muscle forces vary with shoulder position, so future parametric analyses with variable load magnitudes are warranted. Fourth, study results may not be applicable to other three- or four-part proximal humerus fractures. Fifth, material behavior was assumed homogeneous, isotropic, and linearly elastic, which does not capture the anisotropy and tension–compression asymmetry of bone; absolute failure prediction is therefore limited, and emphasis should be placed on relative trends. Sixth, for the humerus at the plate–bone interface, von Mises stress was used as a comparative index to describe local load transfer rather than as a fracture threshold. Seventh, one angle-specific deviation was observed. In the varus model at 70 degrees of traction, the graft maximum principal stress was higher at 30 degrees than at 0 degrees. The mechanism for this difference could not be isolated within the present modeling framework and fixed load magnitude. This finding should be interpreted with caution and examined in future parametric analyses that vary load direction and magnitude as well as construct configuration. At last, screw analysis was limited to the maximum von Mises stress values available for each loading condition. Because screw thread geometry was omitted and fully bonded contacts were applied, potential pull-out or thread-related failure mechanisms could not be assessed. Therefore, the absolute stress values in screws may have been overestimated, and interpretation should be restricted to relative comparisons and the identification of load-bearing screws according to insertion angle.

## 5. Conclusions

In varus and valgus unstable proximal humerus fractures, oblique 30° insertion of the fibular strut reduced plate stresses and, in most traction scenarios, decreased graft stresses compared with vertical 0° insertion, while producing a more dispersed stress distribution on the plate. We suggest that oblique insertion of the fibular strut graft may be an optimal option to sustain multidirectional distraction forces after fracture reduction and fixation.

## Figures and Tables

**Table 1:** Maximum von Mises stresses in the plate, screws, and humerus, and maximum principal stress in the fibular graft, in the varus unstable model (medial wedge-out) (MPa).

	Axial Load				Traction at 70°				Traction at 90°				Traction at 110°			
	Plate	Screw	Humerus	Fibula	Plate	Screw	Humerus	Fibula	Plate	Screw	Humerus	Fibula	Plate	Screw	Humerus	Fibula
Vertical	48.1	148.9	40.6	28.5	268.6	516.5	159.4	121.0	295.8	622.4	187.7	150.7	294.6	656.7	198.1	160.0
Oblique	60.7	134.0	136.6	91.7	26.8	540.6	260.9	186.0	59.4	611.0	322.0	67.5	287.1	618.9	346.6	68.5

**Table 2:** Maximum von Mises stresses in the plate, screws, and humerus, and maximum principal stress in the fibular graft, in the valgus unstable model (lateral wedge-out) (MPa).

	Axial load				Traction at 70°				Traction at 90°				Traction at 110°			
	Plate	Screw	Humerus	Fibula	Plate	Screw	Humerus	Fibula	Plate	Screw	Humerus	Fibula	Plate	Screw	Humerus	Fibula
Vertical	44.6	145.4	47.2	37.0	270.3	494.6	182.8	140.0	296.9	591.5	216.3	175.6	290.8	623.8	221.51	185.1
Oblique	66.9	165.5	174.5	30.5	259.0	589.4	284.1	68.8	289.2	671.3	366.7	79.1	285.1	676.2	369.4	80.7

## Data Availability

The raw data supporting the conclusions of this article will be made available by the authors on request.

## References

[1] Handoll H.H., Brorson S. (2015). Interventions for treating proximal humeral fractures in adults. Cochrane Database Syst. Rev..

[2] Koa J., Fares M.Y., Daher M., Abboud J.A. (2024). Assessment of therapeutic clinical trials for proximal humeral fractures. Clin. Shoulder Elb..

[3] Kim D.Y., Kim T.Y., Hwang J.T. (2020). PHILOS plate fixation with polymethyl methacrylate cement augmentation of an osteoporotic proximal humerus fracture. Clin. Shoulder Elb..

[4] Kim J.Y., Lee J., Kim S.H. (2020). Comparison between MIPO and the deltopectoral approach with allogenous fibular bone graft in proximal humeral fractures. Clin. Shoulder Elb..

[5] Claro R., Barros B., Ferreira C., Ribau A., Barros L.H. (2024). Comparative analysis of proximal humerus fracture management in elderly patients: Complications of open reduction and internal fixation by shoulder surgeons and non-shoulder surgeons-a retrospective study. Clin. Shoulder Elb..

[6] Baron J.A., Barrett J.A., Karagas M.R. (1996). The epidemiology of peripheral fractures. Bone.

[7] Kim S.C., Yoo J.C., Park J.H., Bukhary H., Choi Y.S., Kang K.T., Kim C.H. (2023). Changes in Shoulder Trauma during the COVID-19 Pandemic: A South Korean Survey. Clin. Orthop. Surg..

[8] Patel A.H., Wilder J.H., Ofa S.A., Lee O.C., Iloanya M.C., Savoie F.H., Sherman W.F. (2022). How age and gender influence proximal humerus fracture management in patients older than fifty years. JSES Int..

[9] Iglesias-Rodriguez S., Dominguez-Prado D.M., Garcia-Reza A., Fernandez-Fernandez D., Perez-Alfonso E., Garcia-Pineiro J., Castro-Menendez M. (2021). Epidemiology of proximal humerus fractures. J. Orthop. Surg. Res..

[10] Konrad G., Bayer J., Hepp P., Voigt C., Oestern H., Kaab M., Luo C., Plecko M., Wendt K., Kostler W. (2010). Open reduction and internal fixation of proximal humeral fractures with use of the locking proximal humerus plate. Surgical technique. J. Bone Jt. Surg. Am..

[11] Solberg B.D., Moon C.N., Franco D.P., Paiement G.D. (2009). Locked plating of 3- and 4-part proximal humerus fractures in older patients: The effect of initial fracture pattern on outcome. J. Orthop. Trauma.

[12] Hardeman F., Bollars P., Donnelly M., Bellemans J., Nijs S. (2012). Predictive factors for functional outcome and failure in angular stable osteosynthesis of the proximal humerus. Injury.

[13] Gardner M.J., Boraiah S., Helfet D.L., Lorich D.G. (2008). Indirect medial reduction and strut support of proximal humerus fractures using an endosteal implant. J. Orthop. Trauma.

[14] Bae J.H., Oh J.K., Chon C.S., Oh C.W., Hwang J.H., Yoon Y.C. (2011). The biomechanical performance of locking plate fixation with intramedullary fibular strut graft augmentation in the treatment of unstable fractures of the proximal humerus. J. Bone Jt. Surg. Br..

[15] Osterhoff G., Baumgartner D., Favre P., Wanner G.A., Gerber H., Simmen H.P., Werner C.M. (2011). Medial support by fibula bone graft in angular stable plate fixation of proximal humeral fractures: An in vitro study with synthetic bone. J. Shoulder Elb. Surg..

[16] Hinds R.M., Garner M.R., Tran W.H., Lazaro L.E., Dines J.S., Lorich D.G. (2015). Geriatric proximal humeral fracture patients show similar clinical outcomes to non-geriatric patients after osteosynthesis with endosteal fibular strut allograft augmentation. J. Shoulder Elb. Surg..

[17] Little M.T., Berkes M.B., Schottel P.C., Lazaro L.E., LaMont L.E., Pardee N.C., Nguyen J.T., Helfet D.L., Lorich D.G. (2014). The impact of preoperative coronal plane deformity on proximal humerus fixation with endosteal augmentation. J. Orthop. Trauma.

[18] Matassi F., Angeloni R., Carulli C., Civinini R., Di Bella L., Redl B., Innocenti M. (2012). Locking plate and fibular allograft augmentation in unstable fractures of proximal humerus. Injury.

[19] Neviaser A.S., Hettrich C.M., Beamer B.S., Dines J.S., Lorich D.G. (2011). Endosteal strut augment reduces complications associated with proximal humeral locking plates. Clin. Orthop. Relat. Res..

[20] Tan E., Lie D., Wong M.K. (2014). Early outcomes of proximal humerus fracture fixation with locking plate and intramedullary fibular strut graft. Orthopedics.

[21] Kim D.S., Lee D.H., Chun Y.M., Shin S.J. (2018). Which additional augmented fixation procedure decreases surgical failure after proximal humeral fracture with medial comminution: Fibular allograft or inferomedial screws?. J. Shoulder Elb. Surg..

[22] Lee J.W., Song M.J., Lee S.J., Song H.S., Jung Y.S., Kim H. (2024). Biomechanical comparison between low profile 2.7 mm distal locking hook plate and 3.5 mm distal locking hook plate for acromioclavicular joint injury: A finite element analysis. Injury.

[23] Kim H., Chung Y.G., Jang J.S., Kim Y., Park S.B., Song H.S. (2022). Why locking plates for the proximal humerus do not fit well. Arch. Orthop. Trauma Surg..

[24] Hosoyama T., Kaku N., Pramudita J.A., Shibuta Y. (2024). Comparison of Early Postoperative Stress Distribution around Short and Tapered Wedge Stems in Femurs with Different Femoral Marrow Cavity Geometries Using Finite Element Analysis. Clin. Orthop. Surg..

[25] Tseng J., Huang B., Liang S., Yen K., Tsai Y., Tseng J. (2014). Normal mode analysis of a human fibula. Life Sci. J..

[26] Zhang Q.H., Tan S.H., Chou S.M. (2006). Effects of bone materials on the screw pull-out strength in human spine. Med. Eng. Phys..

[27] Pan X.-H., Chen W.-C., Lin K.-J., Lin K.-P., Tsai C.-L., Wei H.-W. (2019). Effect of semi-rigid locking screws on the stiffness of a fracture-fixation construct: A conceptual finite-element study. Adv. Mech. Eng..

[28] Yang P., Zhang Y., Liu J., Xiao J., Ma L.M., Zhu C.R. (2015). Biomechanical effect of medial cortical support and medial screw support on locking plate fixation in proximal humeral fractures with a medial gap: A finite element analysis. Acta Orthop. Traumatol. Turc..

[29] Fletcher J.W.A., Windolf M., Richards R.G., Gueorguiev B., Varga P. (2019). Screw configuration in proximal humerus plating has a significant impact on fixation failure risk predicted by finite element models. J. Shoulder Elb. Surg..

[30] Hirschmann M.T., Fallegger B., Amsler F., Regazzoni P., Gross T. (2011). Clinical longer-term results after internal fixation of proximal humerus fractures with a locking compression plate (PHILOS). J. Orthop. Trauma.

[31] Launonen A.P., Lepola V., Flinkkila T., Laitinen M., Paavola M., Malmivaara A. (2015). Treatment of proximal humerus fractures in the elderly: A systemic review of 409 patients. Acta Orthop..

[32] Fares M.Y., Singh J., Boufadel P., Cohn M.R., Abboud J.A. (2024). Pyrocarbon hemiarthroplasty and the shoulder: Biomechanical and clinical results of an emerging treatment option. Clin. Shoulder Elb..

[33] Haikal E.R., Fares M.Y., Abboud J.A. (2024). Patient-specific implants in reverse shoulder arthroplasty. Clin. Shoulder Elb..

[34] Gardner M.J., Weil Y., Barker J.U., Kelly B.T., Helfet D.L., Lorich D.G. (2007). The importance of medial support in locked plating of proximal humerus fractures. J. Orthop. Trauma.

[35] Bai L., Fu Z., An S., Zhang P., Zhang D., Jiang B. (2014). Effect of Calcar Screw Use in Surgical Neck Fractures of the Proximal Humerus With Unstable Medial Support: A Biomechanical Study. J. Orthop. Trauma.

[36] Jung W.B., Moon E.S., Kim S.K., Kovacevic D., Kim M.S. (2013). Does medial support decrease major complications of unstable proximal humerus fractures treated with locking plate?. BMC Musculoskelet. Disord..

[37] Zhang L., Zheng J., Wang W., Lin G., Huang Y., Zheng J., Edem Prince G.A., Yang G. (2011). The clinical benefit of medial support screws in locking plating of proximal humerus fractures: A prospective randomized study. Int. Orthop..

[38] Zhang W., Zeng L., Liu Y., Pan Y., Zhang W., Zhang C., Zeng B., Chen Y. (2014). The mechanical benefit of medial support screws in locking plating of proximal humerus fractures. PLoS ONE.

[39] Robinson C.M., Page R.S. (2004). Severely impacted valgus proximal humeral fractures. J. Bone Jt. Surg. Am..

[40] Scheidt M.D., Aiyash S., Salazar D., Garbis N. (2023). Core decompression for early-stage avascular necrosis of the humeral head: Current concepts and techniques. Clin. Shoulder Elb..

[41] DeFranco M.J., Brems J.J., Williams G.R., Iannotti J.P. (2006). Evaluation and management of valgus impacted four-part proximal humerus fractures. Clin. Orthop. Relat. Res..

[42] Walch G., Badet R., Nove-Josserand L., Levigne C. (1996). Nonunions of the surgical neck of the humerus: Surgical treatment with an intramedullary bone peg, internal fixation, and cancellous bone grafting. J. Shoulder Elb. Surg..

[43] Lee S.J., Hyun Y.S., Baek S.H. (2019). Strut Support with Tricortical Iliac Allografts in Unstable Proximal Humerus Fractures: Surgical Indication and New Definition of Poor Medial Column Support. Clin. Shoulder Elb..

[44] Myers D.M., Triplet J.J., Warmoth P.J., Passias B.J., McGowan S.P., Taylor B.C. (2020). Improved Outcomes Using a Fibular Strut in Proximal Humerus Fracture Fixation. Orthopedics.

[45] Tuerxun M., Tuxun A., Zeng L., Wang Q., Chen Y. (2020). Locking Plate Combined With Endosteal Fibular Allograft Augmentation for Medial Column Comminuted Proximal Humeral Fracture. Orthopedics.

[46] Zhang Y.K., Wei H.W., Lin K.P., Chen W.C., Tsai C.L., Lin K.J. (2016). Biomechanical effect of the configuration of screw hole style on locking plate fixation in proximal humerus fracture with a simulated gap: A finite element analysis. Injury.

[47] Kim H., Lee W., Choi S., Kholinne E., Lee E., Alzahrani W.M., Koh K.H., Jeon I.H., Kim S. (2020). Role of Additional Inferomedial Supporting Screws in Osteoporotic 3-Part Proximal Humerus Fracture: Finite Element Analysis. Geriatr. Orthop. Surg. Rehabil..

[48] Chen H., Zhu Z.G., Li J.T., Chang Z.H., Tang P.F. (2020). Finite element analysis of an intramedulary anatomical strut for proximal humeral fractures with disrupted medial column instability: A cohort study. Int. J. Surg..

[49] He Y., Zhang Y., Wang Y., Zhou D., Wang F. (2017). Biomechanical evaluation of a novel dualplate fixation method for proximal humeral fractures without medial support. J. Orthop. Surg. Res..

[50] Chow R.M., Begum F., Beaupre L.A., Carey J.P., Adeeb S., Bouliane M.J. (2012). Proximal humeral fracture fixation: Locking plate construct +/− intramedullary fibular allograft. J. Shoulder Elb. Surg..

[51] Fletcher J.W.A., Windolf M., Grunwald L., Richards R.G., Gueorguiev B., Varga P. (2019). The influence of screw length on predicted cut-out failures for proximal humeral fracture fixations predicted by finite element simulations. Arch. Orthop. Trauma Surg..

